# Structural Characteristic, Strong Antioxidant, and Anti-Gastric Cancer Investigations on an Oleoresin from Ginger (*Zingiber officinale* var. *roscoe*)

**DOI:** 10.3390/foods13101498

**Published:** 2024-05-12

**Authors:** Meichun Chen, Enquan Lin, Rongfeng Xiao, Zuliang Li, Bo Liu, Jieping Wang

**Affiliations:** 1 Institute of Resources, Environment and Soil Fertilizer, Fujian Academy of Agricultural Sciences, Fuzhou 350003, China; cmczjw@163.com (M.C.); neleq@foxmail.com (E.L.); xiaorongfeng@faas.cn (R.X.); fzliubo@163.com (B.L.); 2 Institute of Crop Sciences, Fujian Academy of Agricultural Sciences, Fuzhou 350003, China; lzuliang@163.com

**Keywords:** ginger oleoresin, volatile components, phenolic compounds, antioxidant, cell proliferation and apoptosis

## Abstract

It is known that ginger oleoresin contains various active components and possesses bioactivities. In this study, ginger oleoresin from Chinese ginger (*Zingiber officinale* var. *roscoe*) was extracted using a CO_2_ supercritical fluid extraction method with a 0.52% yield (g/g), based on dry weights. Zingiberene with a content of 51.6 mg/g was the main volatile in the ginger oleoresin. In total, 17 phenolic compounds were identified, and their contents were calculated as 587.54 mg/g. Among them, a new gingertriol was detected in the *Z. officinale*. Antioxidant activity tests showed that the ginger oleoresin and six gingerols exhibited strong scavenging free radical activities, and the zingerone exhibited the strongest antioxidant activity, with IC_50_ values of 11.3 µg/mL for the 2, 2′-diphenyl-1-picrylhydrazyl radical and 19.0 µg/mL for the 2, 2′-amino-di (2-ethyl-benzothiazoline sulphonic acid-6) ammonium salt radical cation, comparable to vitamin C. Ginger oleoresin inhibits HGC-27 human gastric cancer cell proliferation at a rate of 4.05~41.69% and induces cell apoptosis at a rate of 10.4~20.9%. The Western blot result demonstrated that the AKT signaling pathway has the potential mechanism of ginger oleoresin acting on HGC-27 cells. The anticancer potential of the gingerol standards on HGC-27 cells followed the order of 8-gingerol > 6-gingerol > 10-gingerol > zingerone. The different antioxidant and anticancer potentials of the ginger phenolic compounds could be attributed to the presence of hydroxyl groups in the unbranched 1-alkyl chain and the length of carbon side chain. Consequently, ginger oleoresin shows substantial antioxidant and anticancer therapeutic potential and can be used for novel food–drug development.

## 1. Introduction

The fresh rhizome of *Zingiber officinale*, commonly called ginger, is not only an important dietary seasoning in foods due to its unique spicy aroma and taste, but also a traditional Chinese medicine that can treat various diseases efficaciously, such as gastrointestinal discomfort, headache, and common cold [1,2]. *Z. officinale* is a member of the genus *Zingiber*, which has been cultivated in countries enjoying a tropical or subtropical climate including China, India, Jamaica, Malaysia, Nigeria, and others [3]. Ginger is categorized into three types including giant grey ginger (*Z. officinale* var. *roscoe*), small yellow ginger (*Z. officinale* var. *amarum*), and red ginger (*Z. officinale* var. *rubrum*) according to the size and color of the ginger rhizome [4]. In recent years, ginger has gained increasing attention due to its numerous significant biological properties such as anti-microbial, anti-hyperglycemic, and antioxidant [5,6]. The chemical ingredients are considered to be the main contributors to ginger’s bioactive functions [7]. Experiments have proven that CO_2_ supercritical fluid extraction (SFE) is an excellent method to extract bioactive components from fresh ginger, avoiding the drawbacks of chemical solvent extraction, and CO_2_ SFE products (named as essential oils or oleoresins) can be considered as nearly natural foods [8,9].

Ginger oleoresin can scavenge several free radicals, such as 2, 2′-amino-di (2-ethyl-benzothiazoline sulphonic acid-6) ammonium salt radical cation (ABTS·^+^) and 2, 2′-diphenyl-1-picrylhydrazyl radical (DPPH·), improve antioxidant enzyme activities, and reduce the lipid peroxidation of cells to achieve their antioxidant activities [10,11]. Ginger oleoresin can also effectively inhibit the growth of a variety of human tumor cell lines by controlling cell apoptosis and proliferation [12,13]. In addition, anti-inflammatory, anti-obesity, and immunomodulatory activities of ginger oleoresin have also been reported [14].

The above-mentioned biological activities of ginger oleoresin are attributed to various bioactive components derived from ginger [2]. Ginger contains 1–4% essential oil, which is generally composed of phenolic compounds and terpenoids [15]. The phenolic compounds comprise a homologous series of phenolic ketones including gingerols, shogaols, paradols, zingerones, gingerdiones, and gingerdiols, which are responsible for ginger’s pungent odor [7]. Gingerols are biosynthesized from the amino acid phenylalanine; shogaols are generated from the elimination of the 5-hydroxy group in gingerols during heat treatment processing or long-term storage; paradols are the hydrogenated forms of the corresponding shogaols; and gingerdiones are the β-ketone hydroxyl dehydrogenation products from gingerols [16]. The three major phenolic compounds in fresh ginger are 6-gingerol, 8-gingerol, and 10-gingerol, while shogaols and paradols are virtually absent in fresh ginger and are even spicier than gingerols [2]. Some diarylheptanoids have also been found in ginger essential oil [2]. The groups of diarylheptanoids share a β-diketone moiety that includes curcuminoid, bisdemethoxycurcumin, demethoxycurcumin, and curcumin, which can be further classified into linear diarylheptanoid and ring diarylheptanoid compounds [17]. The terpenoids in ginger oil contain monoterpene and sesquiterpene derivatives [15]. Ginger cultivars and the harvesting stage at different maturities influence the yields of oleoresin and the contents of phenolic compounds [18]. Different ginger cultivars exhibit unique aromas, pungencies, appearance, and pharmacological properties. For example, Zaid et al. demonstrated that the essential oil from four cultivars of Malaysian *Z. officinale* Roscoe exhibited different antiproliferative effects on a HeLa cervical cancer cell line due to the differences among their secondary metabolites [19]. The discovery and accurate analysis of new chemical components in ginger oleoresin would promote the development and application of ginger cultivars. Thus, it is of great significance to develop ginger oleoresin as a food–drug from different ginger cultivars for the treatment of various diseases.

In the present study, the active ingredients in Chinese ginger were extracted by the CO_2_ SFE method and then were qualitatively and quantitatively analyzed using gas chromatography–mass spectrometry (GC-MS) and liquid chromatography quadrupole time-of-flight mass spectrometry (LC-QTOF-MS) techniques. The antioxidant and anticancer activities of the ginger extracts were evaluated in vitro. The results of this study could provide useful information for the development and utilization of ginger in new food–medicines.

## 2. Materials and Methods

### 2.1. Chemicals and Reagents

The standards of *α*-curcumene, 6-gingerol, the 6-shogaol/isoshogaol mixture, 8-gingerol, zingerone, 10-gingerol, 10-shogaol, and vitamin C were obtained from Shanghai Yuanye Bio-Technology Co., Ltd. (Shanghai, China). *α*-farnesene was bought from Shanghai Macklin Bio-chemical Technology Co., Ltd. (Shanghai, China). The N-alkane mixture (C_7_~C_40_, HJ894-2017) was obtained from J&K Scientific Co., Ltd. ((Beijing, China)). High-performance liquid chromatography (HPLC)-grade methanol and hexane were purchased from Merck Ind. Ltd. ABTS, DPPH, and potassium persulfate were acquired from Reagent Co., Ltd. (Shanghai, China). The HGC-27 cells, Dulbecco’s Modified Eagle’s Medium (DMEM, containing 3.7 g/L Na_2_CO_3_), fetal bovine serum (FBS), penicillin, and streptomycin were purchased from Wuhan Procell Bio-Technology Co., Ltd. (Wuhan, China). The Cell Counting Kit and Annexin V-fluorescein isothiocyanate/propidium iodide (Annexin V-FITC/PI) Apoptosis Detection Kit were acquired from TransGen Bio-Technology Co., Ltd. (Beijing, China) and Elabscience Biotechnology Co., Ltd. (Wuhan, China), respectively. Antibodies of serine/threonine kinase (Akt), phosphorylated (p)-Akt, extracellular signal-regulated kinase (ERK), and p-ERK were bought from ZenBioScience Technology Co., Ltd. (Chengdu, China). Secondary antibodies of horseradish peroxidase (HRP), goat anti-rabbit immunoglobulin G (IgG) (H + L), HRP goat anti-mouse IgG (H + L), and antibody of anti-β-actin were purchased from ABclonal Biotech Co., Ltd. (Wuhan, China). The CO_2_ SFE apparatus was purchased from Jiangsu Hongbo Machinery Manufacturing Co., Ltd. (Jiangsu, China). Fresh ginger rhizomes (*Zingiber officinale* var. *roscoe*) were collected from plants grown in Shanghang, Fujian, China. This ginger variety (named Taiwan Meat Ginger) was introduced from Taiwan.

### 2.2. Cell Lines

HGC-27 human gastric cancer cells were supplied by Wuhan Procell Bio-Technology Co., Ltd. (Wuhan, China). The cells were cultured in a DMEM medium containing 10% FBS, penicillin (100 U/mL), and streptomycin (100 µg/mL).

### 2.3. CO_2_ Supercritical Fluid Extraction Condition for the Ginger Oleoresin

Ginger powder was obtained from fresh ginger by low-temperature drying and then triturated in a wall breaker. Ginger oleoresin was extracted from the ginger powder using the HB120-50-01 CO_2_ SFE apparatus, and 150 g of ginger powder was put into the extraction kettle. The extraction pressure, extraction temperature, extraction time, separation temperature, and CO_2_ flow rate were set at 30 MPa, 37 °C, 1 h, 54 °C, and 0.3–0.9 L/min, respectively. Finally, the oleoresin was collected from the separation kettle and then stored at −4 °C.

### 2.4. LC-QTOF-MS Analyses of the Ginger Oleoresin

Ginger oleoresin was dissolved in methanol and subjected to LC-QTOF-MS (Agilent 1260 Series HPLC system coupled with Agilent 6520 Series QTOF-MS) for qualitative and quantitative analyses of the phenolic compounds. The data acquisition parameters were set according to the method described by Chen et al. [20]. The linear concentration ranges of the standards of the 6-gingerol, 8-gingerol, and 10-gingerol determined by LC-QTOF-MS were 0.0012~0.66, 0.02~1.45, and 0.0012~0.84 mg/mL, respectively.

### 2.5. GC-MS Analyses of the Volatile Compounds in the Ginger Oleoresin

Ginger oleoresin was dissolved in chloroform for the GC-MS (Agilent 7890A-5975C GC-MS system) analyses. The chromatographic column of HP-5MS (25 m × 0.25 mm i.d.) was selected. The oven temperature gradient was set as follows: (1) held at 80 °C for 3 min; (2) raised to 140 °C at a rate of 20 °C/min and kept at 140 °C for 2 min; (3) increased by 6 °C/min to 230 °C and maintained at 230 °C for 2 min; (4) increased up to 260 °C at a rate of 15 °C and kept at 260 °C for 3 min. Electron impact spectra with full-scanned mode in an m/z range of 35~550 amu were recorded. The ion source temperature and carrier gas helium were set at 230 °C and 18.139 cm/sec, respectively. The analysis of the n-alkane mixture (C_8_~C_40_) was performed under the same GC-MS conditions, and the Kovats retention indices of the detected volatiles were calculated according to the method described by Van den Dool and Kratz [21]. The volatiles’ identification was carried out by comparison of their mass spectra with the NIST14 mass spectral library and further confirmed by the calculated Kovats retention indices with the reference data. The relative contents of all the detected volatiles were calculated by the area normalization method. The standards including the *α*-curcumene, zingarone, and 6-shogaol were selected for qualitative and quantitative analysis. The linear concentration ranges of the standards of the *α*-curcumene, zingarone, and 6-shogaol were 0.00029~0.064, 0.0021~1.2, and 0.008~0.16 mg/mL, respectively.

In addition, the contents of other terpenoids (zingiberene, *α*-farnesene, *β*-bisabolene, and *β*-sesquiphellandrene) in the ginger oleoresin were determined according to the standard curve of the *α*-curcumene solution and defined as milligrams of *α*-curcumene equivalents/gram of ginger oleoresin (mg/g ginger oleoresin). 

### 2.6. Antioxidant Activities of the Ginger Oleoresin

The antioxidant activities of the tested samples (ginger oleoresin, 6-gingerol, 6-shogaol, 8-gingerol, zingerone, 10-gingerol, and 10-shogaol) were detected through the scavenging of the ABTS radical cation (ABTS•^+^) and the DPPH radical (DPPH•). The antioxidant samples were dissolved in methanol in variable concentrations of (10~100 µg/mL). Meanwhile, vitamin C was selected as the positive control sample. Methanol was selected as the negative control sample.

ABTS•^+^ was generated by reacting the ABTS solution (7 mM) with potassium persulfate (2.45 mM) at 28 °C in the dark for 12–16 h. Next, 29 µL of the antioxidant samples and 171 µL of the diluted ABTS•^+^ solution (with an absorbance of 0.70 ± 0.02 at 734 nm) were added to a 96-well microplate and incubated at 28 °C in the dark for 10 min. Then, the absorptions of the mixture of antioxidant samples and diluted ABTS•^+^ solution (A_1_), the mixture of 70% ethanol and diluted ABTS•^+^ solution (A_0_), and the mixture of the antioxidant samples and 70% ethanol (A_2_) were recorded at 734 nm. The radical scavenging activity of ABTS•^+^ by the antioxidant sample was calculated using the following equation: % of ABTS•^+^ inhibition = [1 − (A_1_ − A_2_)/A_0_)] × 100.

The DPPH assays for radical scavenging activities were carried out by adding 0.1 mL samples (10~100 µg/mL) and 0.1 mL 0.1 mM DPPH solution into the 96-microplate. Then, the absorption (A_t_) was detected at 515 nm after the incubation at 28 °C in the dark for 30 min. Methanol was selected as the blank group (A_0_). The DPPH radical scavenging activity was calculated according to the following equation: % of DPPH• inhibition = [(A_0_ − A_t_)/A_0_) × 100%. The antioxidant concentration that inhibited 50% of the free radicals (IC_50_) was calculated using a probit regression model with SPSS 20.0 software.

### 2.7. Anticancer Activities of Ginger Oleoresin

Ginger oleoresin was dispersed in dimethyl sulfoxide and diluted with culture medium into variable concentrations (0~200 µg/mL). Variable concentrations of 6-gingerol (0~50 µg/mL), 8-gingerol (0~5 µg/mL), 10-gingerol (0~5 µg/mL), and zingarone (0~400 µg/mL) standards were prepared. Cell proliferation was measured in vitro on the HGC-27 cell lines after being exposed or not to these extracts using the CCK-8 tests in a 96-well microplate at 37 °C. After 48 h of incubation, the supernatant was removed from each well. Then, 100 µL of cultures containing 10% CCK-8 was added to each well and incubated for another 1 h. The absorption values of the treated (A_1_) and untreated (A_0_) groups were detected at 450 nm. The cell viability (%) was calculated according to the following equation: [(A_1_ − A_c_)/(A_0_ − A_c_)] × 100%, where A_c_ is the absorption value of the culture media.

The cell apoptosis rate was measured by flow cytometry experiments. HGC-27 cells were cultivated in 6-well plates (2 × 10^5^ cells/well) at 37 °C for 24 h. Different concentrations of drugs (100 and 200 μg/mL of ginger oleoresin; 5 μg/mL of 8-gingerol) were added to the cell culture medium. After 48 h of incubation, the cells were collected and washed with phosphate-buffered saline twice. Then, the cells were suspended with 1 × Annexin V binding buffer and incubated with 5 μL Annexin V-FITC and PI reagents at 28 °C in the dark for 15–20 min. Finally, the cells were analyzed using the NovoCyte 1300 Flow Cytometer (ACEA BIO (Hangzhou) Co., Ltd., Hangzhou, China) within 1 h.

The proteins of the HGC-27 cells treated with different concentrations of ginger oleoresin (0~200 μg/mL) for 48 h were extracted. The HGC-27 cells without the ginger oleoresin (only DMEM) treatment were selected as the negative control group. The protein expression level of the HGC-27 was measured using the Western blot method. Initially, the proteins were separated using 10% sodium dodecyl sulfate-polyacrylamide electrophoresis gels and transferred onto polyvinylidene fluoride membranes. Then, the membranes were incubated overnight with anti-Akt (1:500), anti-p-Akt (1:500), anti-ERK (1:800), and anti-p-ERK (1:500) antibodies at 4 °C and then were incubated with HRP-conjugated secondary antibodies (1:4000) for 2 h at 28 °C. Finally, the expression levels of the Akt, p-Akt, ERK, and p-ERK were calculated using Image Lab 6.1 software. The β-actin was used as a control.

### 2.8. Statistical Analyses

All the experiments were carried out three times, and the results were described as the mean ± standard deviation (SD). SPSS 20.0 software was used for the statistical analyses of the data. The Duncan test was used to compare the mean radical scavenging and CCK-8 assays. The difference between the two groups was analyzed by an independent sample *t*-test. *p* < 0.05 and *p* < 0.01 were considered to be significant and remarkably significant, respectively.

## 3. Results and Discussion

### 3.1. Identification of Chemical Compounds of Ginger Oleoresin Obtained by CO_2_ SFE

The ginger oleoresin was extracted by CO_2_ SFE with a 0.52% yield (g/g), based on dry weights. The volatiles from the ginger oleoresin were identified by the GC-MS technique. The total ion chromatogram of the volatiles is shown in Figure S1, and the identification results are summarized in Table 1. A total of 34 volatiles, which accounted for 93.23% of the ginger oleoresin, were identified. It was found that the predominant volatile compounds were 6-shogaol (18.62%), zingiberene (15.34%), zingerone (8.79%), *β*-sesquiphellandrene (8.42%), *α*-curcumene (8.11%), 6-isoshogaol (7.47%), *α*-farnesene (7.09%), and *β*-bisabolene (6.92%). The contents of zingiberene, zingerone, *α*-curcumene, *β*-sesquiphellandrene, *α*-farnesene, and *β*-bisabolene were separately calculated as 51.63 ± 1.29, 49.21 ± 4.43 mg/g, 19.49 ± 0.69, 15.36 ± 0.61, 14.68 ± 0.57, and 6.99 ± 0.14 mg/g of ginger oleoresin by the external standard method. In addition, the total content of 6-shogaol and 6-isoshogaol was calculated as 92.29 ± 7.99 mg/g of ginger oleoresin.

Essential oils from gingers of Eastern Asian origin have been reported to contain a relatively low level of monoterpenes and a high concentration of sesquiterpenes, whereas a preponderance of monoterpenoids in the ginger oil grown in Australia, Malaysia, Madagascar, and other locations has been reported [19,22]. The sesquiterpenes in ginger oleoresin were reported to mainly consist of *α*-curcumene, zingiberene, *α*-farnesene, *β*-bisabolene, and *β*-sesquiphellandrene [19]. These sesquiterpenes were also considered the predominant volatiles in this study. Most of the monoterpenoid-rich oleoresin contained high amounts of α-citral, β-citral, or camphene, and 7.8% camphene was detected in the Ecuador-grown ginger oleoresin [23]; 28.1–70.8% of citral was the main volatile reported in the Australian-grown ginger oleoresin [24]. It was found that a very low amount of α-citral (0.14%) was detected in the China-grown ginger oleoresin in this study.

The phenolic ketones and diarylheptanoids are considered the most common essential oleoresin components in ginger and were analyzed by the LC-QTOF-MS technique in this study. The total ion chromatogram of the ginger oleoresin dissolved in methanol is shown in Figure S2. The identifications of the above compounds in the ginger oleoresin were carried out based on the accurate mass and MS/MS fragmentation by comparison with those of standards or the literature data [25,26,27]. The retention times, MS and MS^2^ spectral characteristics, and identification results are listed in Table 1. The LC-MS/MS fragmentation patterns of deprotonated molecules of compounds 1 (*m*/*z* 373), 2 (*m*/*z* 445), 3 (*m*/*z* 293), 4 (*m*/*z* 291), 5 (*m*/*z* 321), 6 (*m*/*z* 289), 7 (*m*/*z* 349), 8 (*m*/*z* 423), 9 (*m*/*z* 345), 10 (*m*/*z* 277), 11 (*m*/*z* 279), 12 (*m*/*z* 255),13 (*m*/*z* 281), and 14 (*m*/*z* 283) are shown in Figures S3–S7. The above compounds were identified as 5-hydroxy-1, 7, bis (4-hydroxy-3-methoxyphenyl)-3-heptanone (compound **1**), 3, 5-diacetoxy-7-(3, 4-dihydroxyphenyl)-1-(4-hydroxy-3-methoxyphenyl)heptane (compound **2**), 6-gingerol (compound **3**), 1-dehydro-[6]-gingerol (compound **4**), 8-gingerol (compound **5**), 1-dehydro-6-gingerdione (compound **6**), 10-gingerol (compound **7**), dihydrocurcumin derivatives (compound **8**), 1-dehydro-[10]-gingerdione (compound **9**), 1-dehydro-[5]-gingerol (compound **10**), 5-gingerol (compound **11**), 6-(4-hydroxy-3-methoxyphenyl) hexane-1, 2, 4-triol (compound **12**), and 5-gingerdiol (compound **13**), respectively.

Compound 14 eluting at 46.46 min with a deprotonated molecule of *m*/*z* 283 suggested an MW of 284 Da for this compound, and the formula was deduced as C_15_H_24_O_5_. The product ions of *m*/*z* 175 and *m*/*z* 193 were observed (Figure S7a), which were similar to the tandem mass spectrum of gingerol and gingerdiol. The production ion of *m*/*z* 193 probably belongs to the characteristic fragmentation at C4–C5 in gingerol or gingerdiol. According to the fragmentation ions and the accurate molecular weight, the structure of compound 14 was inferred, as shown in Figure S8. This compound was named 8-(4-hydroxy-3-methoxyphenyl) octane-3, 4, 6-triol. It is worth mentioning that compound 14 is a new structure of gingertriol reported in *Z. officinale*. Among them, compounds **3**, **4**, **5**, **6**, **7**, **10**, **11**, **12**, **13**, and **14** are gingerols, compound **9** is a gingerdione, and compounds **1**, **2**, and **8** are diarylheptanoids.

As shown in Table 2, the contents of phenolic ketones and diarylheptanoids in the ginger oleoresin were calculated. Among the phenolic ketones, 6-gingerol, 5-gingerol, 10-gingerol, and 1-dehydro-6-gingerdione were the four main compounds, with contents of 82.93 ± 2.12, 60.84 ± 1.77, 52.63 ± 6.38, and 44.93 ± 0.96 mg/g, respectively. The content of dihydrocurcumin derivatives was calculated as 63.40 ± 2.90 mg/g, which was the predominant diarylheptanoid detected in the ginger oleoresin. Combined with the GC-MS results, the total phenolic compounds detected in the ginger oleoresin were calculated as 587.54 mg/g. The phenolic compounds—6-shogaol, 6-gingerol, 10-gingerol, 5-gingerol, zingerone, 1-dehydro-6-gingerdione, and dihydrocurcumin derivatives—were considered the principal bioactive substances in the ginger oleoresin, which has been reported to have various biological properties including antioxidant, antimicrobial, anticancer, and anti-inflammatory [8].

### 3.2. Antioxidant Activities of Ginger Oleoresin

**Figure 1:**
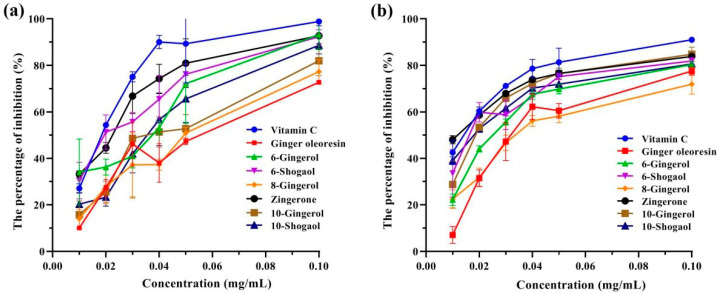
Antioxidant activities of the eight antioxidant samples at different concentrations. (**a**) ABTS; (**b**) DPPH.

Considering the abundant bioactive phenolic compounds in ginger oleoresin, the antioxidant properties of the ginger oleoresin and six phenolic ketone standards were determined by using ABTS •^+^ and DPPH• scavenging assays, with vitamin C as the positive control compound. As shown in Figure 1, all eight antioxidant samples showed dose-dependent antioxidant abilities. The IC_50_ values were calculated for the evaluation of antioxidant potency and are summarized in Table 3. Among the eight antioxidant samples examined, zingerone exhibited the strongest antioxidant activity, with IC_50_ values of 11.3 µg/mL for DPPH• and 19.0 µg/mL for ABTS•^+^, equivalent to the positive control (vitamin C, IC_50_ of 13.0 µg/mL for DPPH• and 17.0 µg/mL for ABTS•^+^). The ginger oleoresin (IC_50_ of 35.7 µg/mL for DPPH• and 48.0 µg/mL for ABTS•^+^) and 8-gingerol (IC_50_ of 36.0 µg/mL for DPPH• and 47.0 µg/mL for ABTS•^+^) exhibited similar antioxidant capacities, which were significantly weaker than the other four samples (IC_50_ of 16.7~25.0 µg/mL for DPPH• and 20.7~38.7 µg/mL for ABTS•^+^).

The phenolic ketones behaved as efficient electron or hydrogen donors, which are considered excellent radical scavengers [28]. The antioxidant potential of phenolic ketone was shown to be closely correlated with the compound structure [29]. The zingerone and shogaol in the ginger were shown to be the thermal dehydrated forms of gingerol [2]. The zingerone, gingerols, and shogaols shared the same basic structural unit: a 1, 2, 4-trisubstituted benzene ring containing a 3-methoxy group, 4-hyderoxyl group, and an unbranched 1-alkyl chain (length from 4, 6, 8, 10, or 12 carbons), as shown by the dotted red lines in Figure S9. The gingerols contain a hydroxyl group at C5 in the alkyl chain, while the shogaols contain a double bond at C4–C5. Our results showed that the relative scavenging potential for stable free radicals was in the order of zingerone > 6-shogaol ≥ 10-shogaol, which indicated that the longer unbranched 1-alkyl chain length exhibited a negative effect in antioxidant properties. It was noted that the shogaols exhibited stronger scavenging free radical activities than the gingerols, which demonstrated that the double bond at the C4–C5 moiety was more important than a hydroxyl group at C5 in the structure. This result was consistent with the previously published reports. As an example, Dugasani et al. demonstrated that 6-shogaol exhibited the strongest scavenging activity among the tested four substances—6-shogaol, 6-gingerol, 8-gingerol, and 10-gingerol [30]. Zingerone had been reported as a good scavenger acting on the peroxyl radicals, yet it displayed a weak inhibitory ability on the peroxidation of phospholipid liposomes [31]. In the present study, we found that the zingerone also displayed excellent scavenger radical ability on DPPH• or ABTS•^+^, comparable to vitamin C. These results indicated that zingerone could be considered an excellent radical scavenger.

### 3.3. In Vitro Anticancer Activity

**Figure 2:**
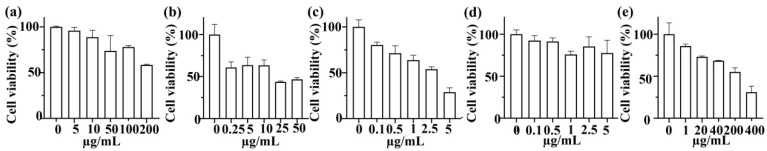
The cytotoxic effects of ginger oleoresin (**a**), 6-gingerol (**b**), 8-gingerol (**c**), 10-gingerol (**d**), and zingerone (**e**) on human tumor HGC-27 cell growth at different concentrations for 48 h. Cell viability was examined by CCK-8 assay. All the data were obtained from three independent experiments and expressed as the mean ± SD.

The phenolic ketones in ginger have been shown to have good inhibitory effects on various tumor cell lines. In this study, the inhibitory effects of ginger oleoresin and four phenolic ketones of ginger (6-gingerol, 8-gingerol, 10-gingerol, and zingerone) against HGC-27 lines were evaluated by using the CCK-8 method. The ginger oleoresin showed cytotoxic effects against HGC-27 cells, with an inhibition rate of 4.05%~41.69% at concentrations of 5~200 µg/mL (Figure 2a). The cytotoxic activities of 6-gingerol, 8-gingerol, 10-gingerol, and zingerone on HGC-27 cells were further examined, as shown in Figure 2b–e. The results showed that the cytotoxic activity of 5 µg/mL of 10-gingerol was nearly identical to that of 20 µg/mL of zingerone, indicating that the 10-gingerol displayed stronger cytotoxic activity than zingerone. Furthermore, the 10-gingerol demonstrated weaker cytotoxic activity than 6-gingerol at the same concentration of 5 µg/mL. Thus, the anticancer effect was in the order of 8-gingerol (IC_50_ = 2.0 µg/mL) > 6-gingerol (IC_50_ = 31.4 µg/mL) > 10-gingerol > zingerone (IC_50_= 165.3 µg/mL). Interestingly, the 8-gingerol displayed the strongest inhibiting activity on the proliferation of HGC-27 cells. These results suggest that the influence of the presence of hydroxyl groups in the unbranched 1-alkyl chain is superior to that of side-chain length in displaying anti-proliferative activities.

Some of the literature reported that 10-gingerol exhibited stronger inhibitory effects than 6-gingerol and 8-gingerol on breast cancer cells or liver cancer cells [32]. However, our results revealed that 8-gingerol and 6-gingerol display stronger inhibition activities than 10-gingerol on the proliferation of gastric cancer cells. Among the above compounds, 6-gingerol was reported to exert suppressive effects on the proliferation of various cancer cells, such as lung, liver, oral, cervical, gastrointestinal, and colon cancers [33]. Luo et al. demonstrated that 6-gingerol decreased the viability of HGC-27 cells in a dose-dependent mode, with an IC_50_ value of 386.3 µM (113.7 µg/mL) at 48 h. In addition, 8-gingerol is the main active component in ginger [34]. Yet, there is little research concerning the activity of 8-gingerol against cancer. Hu et al. demonstrated that 8-gingerol suppresses proliferation, with an IC_50_ value of 77.4 µM at 48 h, and induced apoptosis of the human colorectal cancer HCT116 cells [35]; Su et al. revealed that 8-gingerol inhibits the proliferation of the human liver cancer HepG2 cells, with an IC_50_ value of 20 µM [32]. This study is the first to report the strong anti-proliferative effect of 8-gingerol on gastric cancer cells, with a low IC_50_ value of 6.2 µM (2 µg/mL).

**Figure 3:**
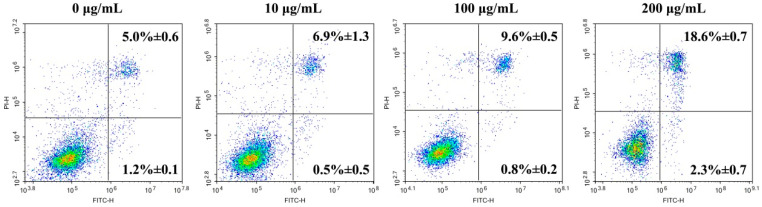
Apoptotic effects of ginger oleoresin on HGC-27 cells for 48 h examined by flow cytometry. The upper right quadrant represents late apoptotic cells, and the lower right quadrant represents early apoptotic cells. All the experiments were performed in triplicate and expressed as the mean ± SD.

The HGC-27 cells under treatment with different concentrations of ginger oleoresin for 48 h were further stained with Annexin V-FITC/PI. The results of flow cytometry indicated that the apoptotic rate of HGC-27 cells increased along with the increased concentration of ginger oleoresin. There was no significant difference in the apoptotic rate of HGC-27 cells between the 10 μg/mL treated group and the negative control group. When exposed to 100~200 μg/mL of ginger oleoresin, the HGC-27 cells exhibited apoptotic rates of 10.4~20.9%. It was found that the late apoptotic cell rate was obviously raised with the increase in ginger oleoresin concentrations, while the early apoptotic cell rate was not (Figure 3), indicating that ginger oleoresin could induce late apoptosis of HGC-27 cells. The apoptotic effect of 8-gingerol on HGC-27 cells was further examined because it had the strongest anti-proliferation activity. The apoptotic rate of HGC-27 cells treated with 8-gingerol (5 µg/mL) was calculated as 12.19 ± 1.22% (Figure S10). Combined with these data, it could be inferred that ginger oleoresin and 8-gingerol inhibited the proliferation of HGC-27 cells partially by inducing apoptosis.

**Figure 4:**
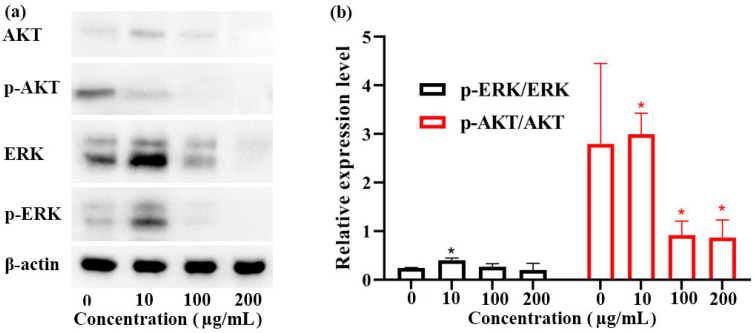
Western blot of AKT, p-AKT, ERK, and p-ERK in HGC-27 cells (**a**); the relative protein expression levels of p-AKT/AKT and p-ERK/ERK in HGC-27 cells (**b**). * *p* < 0.05 vs. negative control group (0 µg/mL).

The ERK and AKT pathways are two important signaling pathways relating to cell proliferation, apoptosis, and migration [36]. After treatment with 0~200 µg/mL of ginger oleoresin for 48 h, the expression levels of total AKT, p-AKT, total ERK, and p-ERK were evaluated by Western blot. It was found that the expression levels of AKT, p-AKT, ERK, and p-ERK proteins were down-regulated by treatment with 100~200 µg/mL of ginger oleoresin (Figure 4a). Compared with the negative control group, the relative expression levels of p-AKT/AKT were significantly down-regulated by treatment with 100~200 µg/mL of ginger oleoresin, but those of p-ERK/ERK were not (Figure 4b). These results demonstrate that ginger oleoresin inhibited proliferation and induced apoptosis of HGC-27 cells in vitro by modulating the AKT signaling pathway.

## 4. Conclusions

Ginger has been widely recorded as a traditional natural medicine for the treatment of numerous diseases due to its various active compounds. In this study, ginger oleoresin was extracted by CO_2_ SFE. The predominant volatiles in the ginger oleoresin were sesquiterpenes including zingiberene, α-curcumene, β-sesquiphellandrene, α-farnesene, and β-bisabolene. Furthermore, 17 phenolic compounds, including gingerols, gingerdiones, and diarylheptanoids, were identified: 6-shogaol, 6-gingerol, 10-gingerol, 5-gingerol, zingerone, 1-dehydro-6-gingerdione, and dihydrocurcumin derivatives were considered to be the major phenolic constituents in the ginger oleoresin. Most importantly, 8-(4-hydroxy-3-methoxyphenyl) octane-3, 4, 6-triol was a new gingertriol reported in *Z. officinale*.

The antioxidant activities of ginger oleoresin and its main phenolic compounds were evaluated. The results showed that ginger oleoresin exhibited similar antioxidant capacities to 8-gingerol, and zingerone displayed the strongest antioxidant activity due to its shorter unbranched 1-alkyl chain length. Meanwhile, it was found that the shogaols demonstrated stronger scavenging free radical activities than the gingerols, which could be attributed to the presence of a double bond at the C4–C5 moiety. Ginger oleoresin exerted anti-cancer effects on HGC-27 cells via inhibiting proliferation and inducing apoptosis, which could be attributed to the modulation of the AKT signaling pathway. The anticancer potential of four phenolic compounds (8-gingerol > 6-gingerol > 10-gingerol > zingerone) suggested that the effect of hydroxyl groups in the unbranched 1-alkyl chain was more dominant than that of the side-chain length in terms of anti-proliferative properties. Importantly, this is the first study to report the strong anti-proliferative effect of 8-gingerol on gastric cancer cells, with a low IC_50_ value. These results offer some basis for the application and development of ginger oleoresin as a combination food–drug.

## Figures and Tables

**Table 1:** The GC-MS identification results of the ginger oleoresin obtained by supercritical CO_2_.

Retention Time/min	Compound Identification	Molecular Formula	KI ^a^	KI ^b^	Relative Content (%)
7.90	Decanal	C_10_H_20_O	1195	1208.8	0.56 ± 0.05
8.97	α-citral	C_10_H_16_O	1269	1275.7	0.14 ± 0.01
9.30	2-Undecanone	C_11_H_22_O	1294	1296.3	0.12 ± 0.06
10.98	*α*-Copaene	C_15_H_22_	1376	1391.7	0.58 ± 0.07
11.23	*β*-Elemene	C_15_H_22_	1407	1405.3	0.57 ± 0.02
11.37	Trans-*α*-Bergamotene	C_15_H_22_	1438	1413.2	0.36 ± 0.04
11.85	*β*-ylangene	C_15_H_22_	1423	1438.6	0.18 ± 0.03
12.00	*γ*-Elemene	C_15_H_22_	1418	1446.4	0.63 ± 0.08
12.28	*β*-trans-bergamotene	C_15_H_22_	1490	1461.4	0.21 ± 0.14
12.35	*β*-Farnesene	C_15_H_22_	1459	1465.2	0.52 ± 0.11
12.65	Alloaromadendrene	C_15_H_22_	1457	1481.2	0.08 ±0.09
12.87	*α*-curcumene	C_15_H_22_	1483	1492.8	8.11 ±0.64
13.12	Zingiberene	C_15_H_22_	1493	1506.2	15.34 ± 1.08
13.27	*α*-Farnesene	C_15_H_22_	1507	1514	7.09 ± 0.51
13.37	*β*-Bisabolene	C_15_H_22_	1506	1519.3	6.92 ± 0.53
13.58	(+)-epi-Bicyclosesquiphellandrene	C_15_H_22_	1521	1530.4	0.07 ± 0.14
13.69	*β*-Sesquiphellandrene	C_15_H_22_	1525	1536	8.42 ± 0.53
13.80	(E)-*γ*-Bisabolene	C_15_H_22_	1533	1542.2	0.24 ± 0.03
14.13	Dodecanoic acid	C_12_H_24_O_2_	1570	1559.6	0.16 ± 0.01
14.75	Cetene	C_16_H_32_	1587	1592.2	1.56 ± 0.13
14.88	Hexadecane	C_16_H_34_	1600	1599.1	0.38 ± 0.03
16.07	Zingerone	C_11_H_14_O_3_	1653	1663.1	8.79 ± 0.27
16.73	Heptadecane	C_17_H_36_	1700	1698.1	0.23 ± 0.09
18.42	1-Octadecene	C_18_H_36_	1795	1791.9	0.57 ± 0.04
19.86	Phthalic acid, isobutyl octyl ester	C_20_H_30_O_4_	1868	1874.6	1.04 ± 0.06
21.82	(E)-1-(6,10-Dimethylundec-5-en-2-yl)-4-methylbenzene	C_20_H_32_	1991	1991.1	1.26 ± 0.13
23.95	6-Methyl-4,6-bis(4-methylpent-3-en-1-yl)cyclohexa-1,3-dienecarbaldehyde	C_20_H_30_O	2113.4	2120.6	1.33 ± 0.17
24.70	Cyclohexene, 4-(4-ethylcyclohexyl)-1-pentyl-	C_19_H_34_	NA	2161.4	0.14 ± 0.04
25.14	2-Methyl-3-(3-methyl-but-2-enyl)-2-(4-methyl-pent-3-enyl)-oxetane	C_15_H_26_O	NA	2185.5	0.55 ± 0.06
25.26	1-Docosene	C_22_H_44_	NA	2192	0.32 ± 0.02
26.53	6-Isoshogaol	C_17_H_24_O_3_	2231.7	2259.7	7.47 ± 0.43
27.56	6-Shogaol	C_17_H_24_O_3_	2294	2319.2	18.62 ± 2.91
28.60	Z-12-Pentacosene	C_25_H_50_	NA	2393.3	0.29 ± 0.18
30.16	Pentacosane	C_25_H_52_	2500	2498	0.38 ± 0.14

Note: KI ^a^ means the Kovats retention indices in the reference. KI ^b^ means the calculated Kovats retention indices in this study. NA means it was not found in the literature.

**Table 2:** The LC-MS/MS identification results of ginger oleoresin.

Compound	Retention Time (min)	MS	MS^2^	Identification	Content (mg/g Ginger Oleoresin)
1	29.20	373	179, 193, 165, 121	5-hydroxy-1,7, bis (4-hydroxy-3-methoxyphenyl)-3-heptanone	23.67 ± 0.33
2	33.16	445	385, 325, 355	3,5-diacetoxy-7-(3, 4-dihydroxyphenyl)-1-(4-hydroxy-3-methoxyphenyl)heptane	4.88 ± 0.18
3	33.59	293	193, 135, 178, 100, 275	6-gingerol	82.93 ± 2.12
4	34.30	291	191, 176, 135, 162	1-dehydro-[6]-gingerol	17.04 ± 0.19
5	37.01	321	289, 274, 193, 175, 160, 149, 134, 127	8-gingerol	11.94 ± 0.37
6	37.75	289	274, 219, 191, 175, 160, 149, 134	1-dehydro-6-gingerdione	44.93 ± 0.96
7	38.90	349	331, 289, 274, 193, 175, 155, 149, 134	10-gingerol	52.63 ± 6.38
8	40.09	423	329, 149, 177, 287, 133	dihydrocurcumin derivatives	63.40 ± 2.90
9	42.06	345	277, 149, 195, 175, 163, 134	1-dehydro-[10]-gingerdione	19.93 ± 1.59
10	42.59	277	259, 205, 195, 175, 149, 134	1-dehydro-[5]-gingerol	10.12 ± 1.08
11	43.26	279	195, 175, 149	5-gingerol	60.84 ± 1.77
12	43.80	255	237	6-(4-hydroxy-3-methoxyphenyl)hexane-1,2,4-triol	17.16 ± 1.36
13	44.58	281	275, 149, 134	5-gingerdiol	9.93 ± 2.16
14	46.46	283	175, 193	8-(4-hydroxy-3-methoxyphenyl)octane-3,4,6-triol	4.16 ± 0.35

Note: The contents of 6-gingerol, 8-gingerol, and 10-gingerol in the ginger oleoresin were calculated based on the corresponding standard curve, while others were calculated according to the standard curve of 6-gingerol solution. The contents of the detected compounds were defined as milligrams of standard equivalents/gram of ginger oleoresin.

**Table 3:** Antioxidant activities of the eight antioxidant samples expressed as 50% inhibition (IC_50_).

No.	Compounds	Mass Weight	Half Maximal Inhibitory Concentration (IC_50_, µg/mL)	
			ABTS	DPPH
1	Vitamin C	176	17.0 ± 0.0 *^e^*	13.0 ± 2.0 *^de^*
2	Ginger oleoresin		48.0 ± 0.0 *^a^*	35.7 ± 3.5 *^a^*
3	6-gingerol	294	27.3 ± 5.1 *^cd^*	25.0 ± 1.0 *^b^*
4	6-shogaol	276	20.7 ± 2.3 *^de^*	17.7 ± 5.5 *^cd^*
5	8-gingerol	322	47.0 ± 6.1 *^a^*	36.0 ± 4.4 *^a^*
6	Zingerone	194	19.0 ± 1.0 *^d^^e^*	11.3 ± 0.6 *^e^*
7	10-gingerol	350	38.7 ± 10.1 *^b^*	19.0 ± 2.6 *^c^*
8	10-shogaol	332	33.3 ± 1.5 *^bc^*	16.7 ± 1.2 *^cde^*

Note: Data are presented as mean ± standard deviation (*n* = 3). Different letters in the same column indicate that the difference between the grades was significant in the Duncan test (*p* < 0.05). Vitamin C was used as a positive control.

## Data Availability

The data underlying this article are available in the article and its online Supplementary Material, further inquiries can be directed to the corresponding author.

## Supplementary Materials

The following supporting information can be downloaded at: https://www.mdpi.com/article/10.3390/foods13101498/s1, Figure S1: The GC-MS total ion chromatogram (TIC) of volatile compounds in ginger oleoresin.; Figure S2: The LC-MS total ion chromatogram (TIC) of ginger oleoresin dissolved in methanol; Figure S3: The LC-MS/MS fragmentation patterns of deprotonated molecule of compounds **3**, **5** and **7** (*m*/*z* 293, *m*/*z* 321 and *m*/*z* 349, respectively); Figure S4: The LC-MS/MS fragmentation patterns of deprotonated molecule of compounds **4**, **6** and **10** (*m*/*z* 291, *m*/*z* 289 and *m*/*z* 277, respectively); Figure S5: The LC-MS/MS fragmentation patterns of deprotonated molecule of compounds **11**, **13** and **9** (*m*/*z* 279, *m*/*z* 281 and *m*/*z* 345, respectively); Figure S6: The LC-MS/MS fragmentation patterns of deprotonated molecule of compounds **1**, **2** and **8** (*m*/*z* 373, *m*/*z* 445 and *m*/*z* 423, respectively); Figure S7: The LC-MS/MS fragmentation patterns of deprotonated molecule of compounds **14** and **12** (*m*/*z* 283 and *m*/*z* 255, respectively); Figure S8: The structures of compounds **14**; Figure S9: The structures of zingerone, gingerols and shogaols; Figure S10: Apoptotic effect of 8-gingerol on HGC-27 cells for 48 h was examined by flow cytometry. The upper right quadrant represented late apoptotic cells and the lower right quadrant represented early apoptotic cells. All experiments were performed in triplicates and expressed as mean ± SD.
