# Supplementary material for: Structural Characteristic, Strong Antioxidant, and Anti-Gastric Cancer Investigations on an Oleoresin from Ginger (Zingiber officinale var. roscoe)
Source: Foods. 2024 May 12;13(10):1498. doi: 10.3390/foods13101498 (PMC11119446; doi:10.3390/foods13101498)
Supplement: Supplementary file 1 [file foods-13-01498-s001.zip › foods-3010373-supplementary.pdf]

Structural Characteristic, Strong Antioxidant, and Anti-Gastric Cancer Investigations on an Oleoresin from Ginger (*Zingiber officinale* var. *roscoe*)

Meichun Chen[1], Enquan Lin[1], Rongfengxiao[1], Zuliang Li[2], Bo Liu[1], Jieping Wang[1*]

[1] Institute of Resources, Environment and Soil Fertilizer, Fujian Academy of Agricultural Sciences, Fuzhou, 350003, China.

[2] Institute of Crop Sciences, Fujian Academy of Agricultural Sciences, Fuzhou, 350003, China.

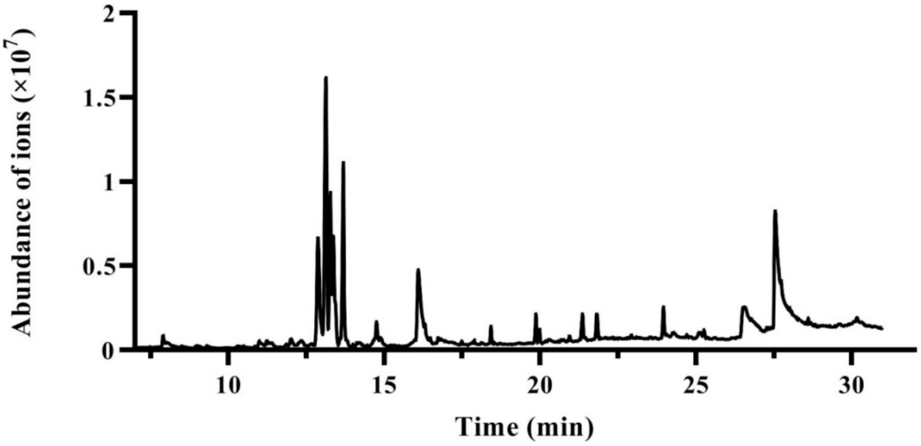

Figure S1. The GC-MS total ion chromatogram (TIC) of volatile compounds in ginger oleoresin.

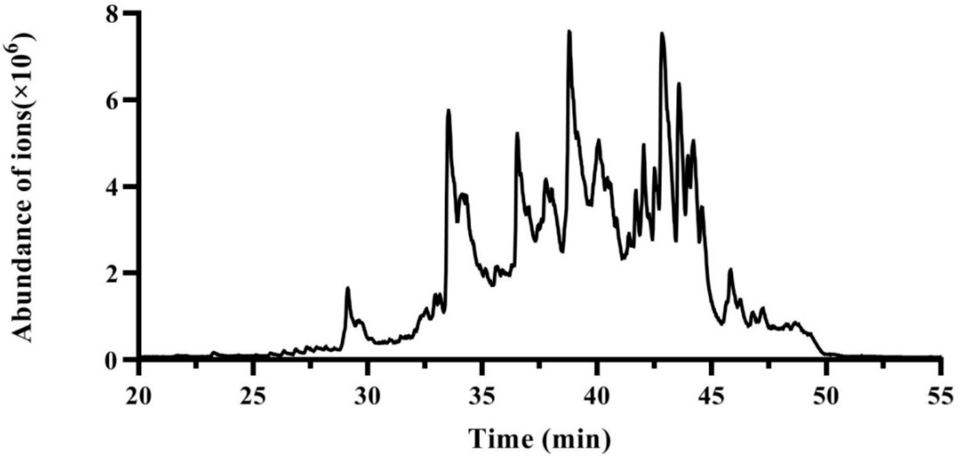

Figure S2. The LC-MS total ion chromatogram (TIC) of ginger oleoresin dissolved in methanol

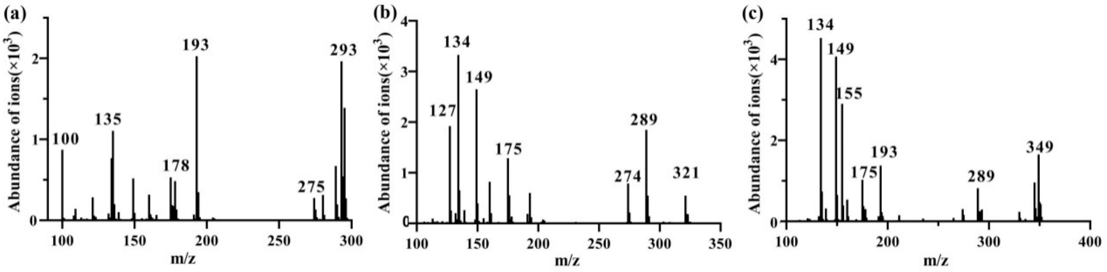

Figure S3. The LC-MS/MS fragmentation patterns of deprotonated molecule of compounds 3, 5 and 7 (m/z 293, m/z 321 and m/z 349, respectively)

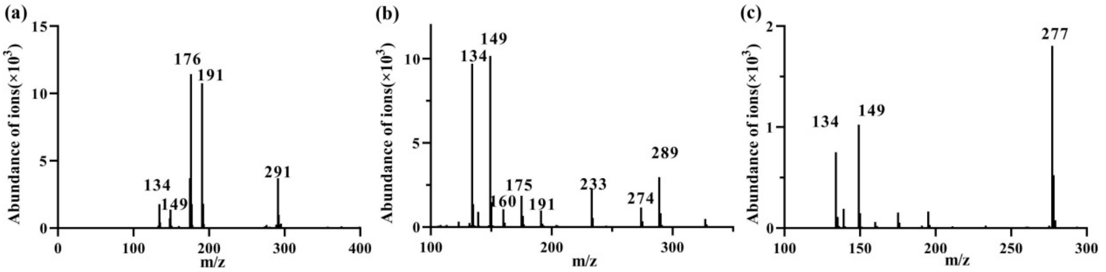

Figure S4. The LC-MS/MS fragmentation patterns of deprotonated molecule of compounds 4, 6 and 10 (m/z 291, m/z 289 and m/z 277, respectively)

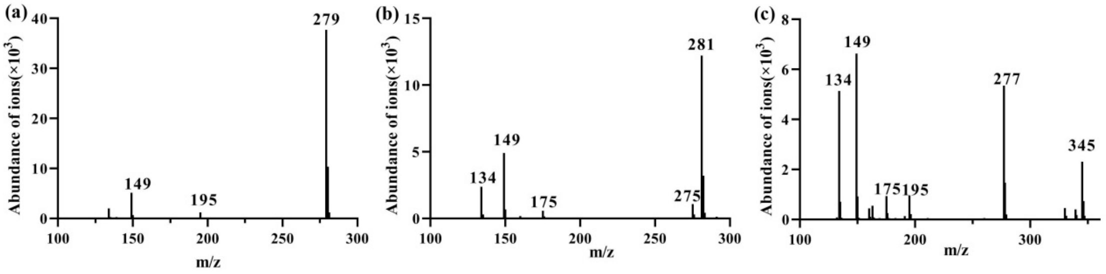

Figure S5. The LC-MS/MS fragmentation patterns of deprotonated molecule of compounds 11, 13 and 9 (m/z 279, m/z 281 and m/z 345, respectively)

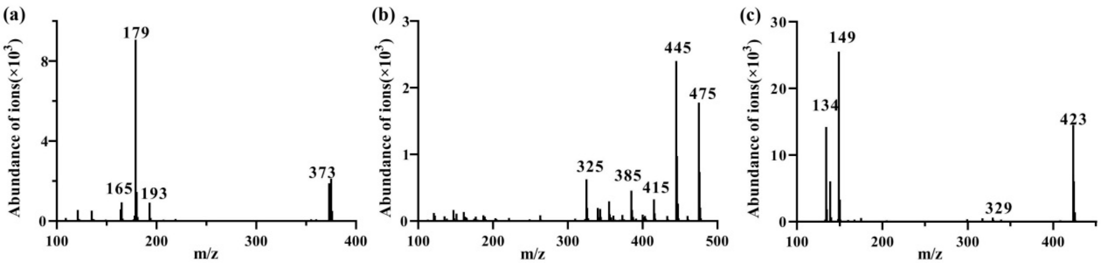

Figure S6. The LC-MS/MS fragmentation patterns of deprotonated molecule of compounds 1, 2 and 8 (m/z 373, m/z 445 and m/z 423, respectively)

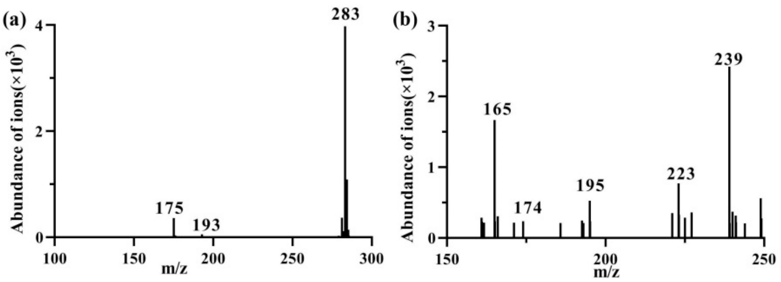

Figure S7. The LC-MS/MS fragmentation patterns of deprotonated molecule of compounds 14 and 12 (m/z 283 and m/z 255, respectively)

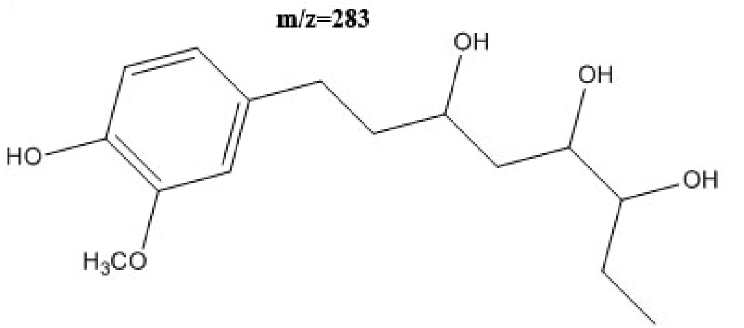

Figure S8. The structures of compounds 14.

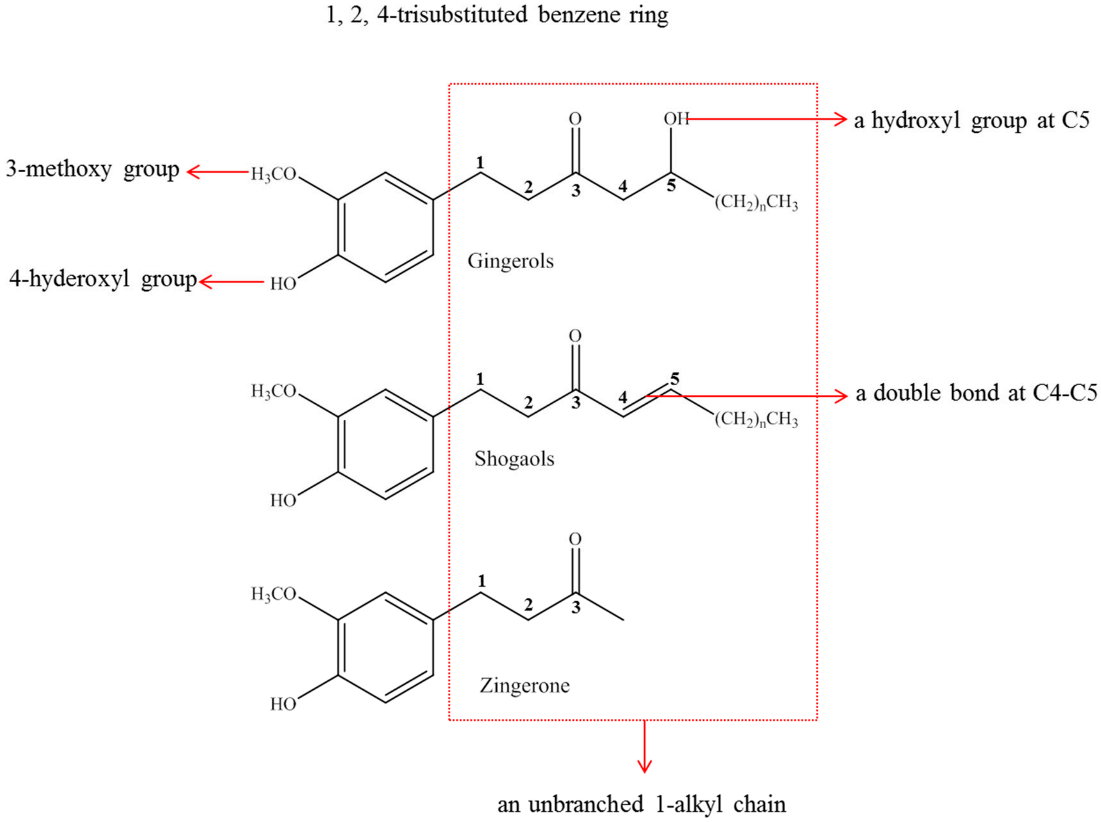

Figure S9. The structures of zingerone, gingerols and shogaols.

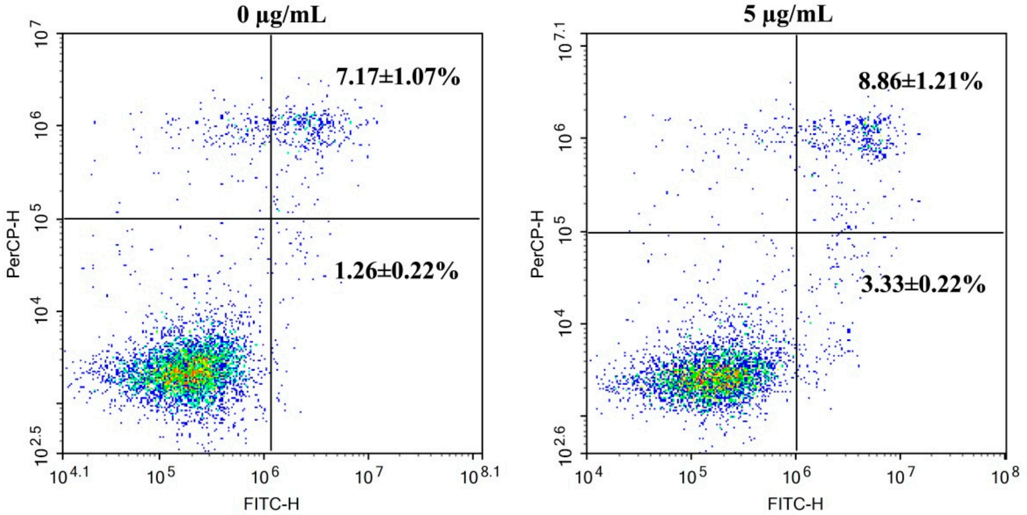

Figure S10.Apoptotic effect of 8-gingerol on HGC-27 cells for 48 h was examined by flow cytometry. The upper right quadrant represented late apoptotic cells and the lower right quadrant represented early apoptotic cells. All experiments were performed in triplicates and expressed as mean ± SD.
